# The dimensionality of morphological awareness in reading comprehension among Chinese early adolescent readers

**DOI:** 10.1371/journal.pone.0276546

**Published:** 2022-10-27

**Authors:** Haomin Zhang, Jiexin Lin, Xi Cheng, Jiefang Li

**Affiliations:** 1 The Psycholinguistics Lab, School of Foreign Languages, East China Normal University, Shanghai, China; 2 Department of Foreign Languages and Literatures, Tsinghua University, Beijing, China; 3 Michtom School of Computer Science, Brandeis University, Waltham, Massachusetts, United States of America; University of Birmingham, UNITED KINGDOM

## Abstract

Recent research has included multiple measures of morphological awareness to address the dimensionality of morphological construct in the context of modeling skilled reading. However, a majority of studies have an Anglocentric focus. The current study aims to extend the previous studies to logographic learners by evaluating the dimensionality of morphological awareness in higher-order reading comprehension among Chinese adolescent readers. A total of 686 early adolescent students (339 fifth-grade students and 347 sixth-grade students) participated in the study. They completed a series of morphological awareness measurements (morpheme recognition, morpheme discrimination, and compound structure awareness), vocabulary knowledge, lexical inference and reading comprehension. By testing three alternative path models, the study showed that morphological awareness and vocabulary knowledge were best represented as parallel covariates in predicting Chinese reading comprehension. More important, the study highlighted the mediator of lexical inference in associating morphological awareness, vocabulary knowledge and reading comprehension among Chinese readers. Empirical findings suggest that morphological awareness and vocabulary knowledge seem to be under a unitary construct in logographic reading acquisition and that word-meaning inference ability connects the path between morphological awareness and reading comprehension. These findings contribute to the complexity in the conceptualization of Chinese morphological awareness and reading instruction by examining the ways in which the morphological construct supports higher-order reading development.

## Introduction

Morphological awareness, “conscious awareness of the morphemic structure of words and their ability to reflect on and manipulate that structure” [1], has been identified as a crucial precursor in reading comprehension [2–5]. In its support of literacy development, morphological awareness has been regarded as multidimensionally embracing the complex interplay of reading-related skills [2]. Recently, interest has increased to address the underlying nature of the morphological awareness (MA) construct by incorporating vocabulary knowledge in modeling skilled reading in alphabetic orthographies (e.g., [6, 7]). Findings were mixed with regard to the relation between morphological awareness and vocabulary. Spencer et al. [8] first proposed a unidimensional model that encompassed morphological awareness and vocabulary knowledge; while follow-up studies pinpointed the multidimensionality of morphological awareness separable from vocabulary knowledge and further confirmed their predictive validities in reading (e.g., [6, 9]). However, much prior work has focused on alphabetic languages such as English [8, 10] or partial phonemic/abjad like Arabic [7], while ignoring more logographic languages. It is of particular importance to move beyond English to evaluate the dimensionality of morphological awareness in logographic scripts like Chinese. Unlike the morphophonemic language of English, Chinese is a morphosyllabic system in which one morpheme usually corresponds to one syllable in spoken form [11–13]. Thus, the first aim of the current study was to explore the dimensionality of morphological awareness by tapping three sets of distinctions among Chinese lexical morphological awareness including morphological discrimination, recognition, compound structure awareness as well as its association with vocabulary knowledge. In other words, we are interested in disentangling whether morphological awareness and vocabulary knowledge are separate constructs or the same underlying construct among Chinese early adolescent readers. Such a theoretical distinction of the dimensionality into morphology construct helps demystify the complexity of morphological awareness in Chinese.

In addition to unpacking the dimensionality of morphological awareness in Chinese, we also see a need to disentangle the manners in which morphological awareness influences higher-order reading comprehension. Growing empirical literature speaks strongly to the universal presence of a pivotal role for morphology in literacy development [14–16], with mixed findings constructing multiple plausible paths. Some empirical evidence indicated that morphological awareness directly predicted reading comprehension across different languages [3, 17, 18]. Nevertheless, prior explorations mostly stemmed from an Anglocentric focus and what we would expect for English may not apply to other scripts with a more transparent orthography. In the context of Chinese learners of English, recent evidence highlighted an indirect pathway from morphological awareness to reading comprehension via vocabulary and lexical inference [19, 20]. Thus, in what follows, we explore more evidence of the precise pathways for morphological awareness to impact logographic literacy development by testing a plausible intermediary candidate such as lexical inferencing ability. It is also important to note that the participating adolescent readers from 4^th^ and 5^th^ grade levels are supposed to have moved from “learning to read” to “reading to learn” [21]. Learners at this stage have likely consolidated grapheme-phoneme correspondence rules in word reading to support higher-order reading, and they can gain new knowledge and apply multiple reading strategies during reading. Learners at 15–17 years old can react to multiple viewpoints critically and comprehend layers of facets. As they grow more mature, they start reading more difficult materials for their own needs, and their reading becomes more rapid and efficient [21]. Therefore, the current study intends to demystify how the Chinese morphological construct works in reading during such a critical stage of literacy development.

### Morphological awareness in Chinese

Chinese provides a fascinating window into the role of dimensionality of morphological awareness in reading comprehension given its unique features of morphological structure at the lexical level. Chinese is a morphosyllabic system in which Chinese orthography encodes characters as graphic symbols and each morpheme corresponds to a single character with a syllable, with the exception of several combinations of a few morphemes comprised of two characters [13]. There are two levels of morphological awareness in Chinese: the lexical compounding at the word level; and the internal structure of the character at the character level (submorphemic unit including radicals and phonetic components) [22]. Since children’s ability in utilizing submorphemic units to infer meanings of unknown characters has grown to mature as early as in the 3rd grade [23] and much empirical data have highlighted the uniqueness of lexical compounding awareness in Chinese reading [14–16], the current study, therefore, addressed the issue of morphological awareness at the lexical level, which is defined as the ability to understand specific morphemes and manipulate the morphological structure rules of constructing Chinese compound words from single characters/morphemes [24, 25]. Additionally, unlike English where inflections and derivations are highly productive in new word formation, compounding of characters is the principal rule for Chinese word formation [26] as more than 70% of Chinese words are compounds consisting of two or more morphemes [27]. Furthermore, the discrepancy between lexical compounding structures also underpins the necessity to advance the role of MA in logographic scripts beyond Anglocentric scripts. In English, the subordinate structure seems to be predominant in lexical compounding [15]. It is often the case in English subordinate compounding that the first morpheme modifies the second, in which the second morpheme carries more weight in constituting the meaning of the compound. For instance, “room” is the important head morpheme denoting the syntactic (as a Noun) and semantic information (a building with walls, floor, ceiling) in “bathroom”, and “bath” as a modifier narrows the semantic meaning, specifying what kind of the room it is. Hence, the second morpheme is more important for constructing the meaning of the whole compound word. In contrast, Chinese compounding structures are relatively rich including coordinative, subordinate, subject-predicate, verb-object, and verb/adjective-complement [15]. In coordinative structures in Chinese, which can hardly be found in English (with a few exceptions like in-and-out), each constituting morpheme is equally prominent for the meaning of the whole compound word (e.g., the meaning of the compound “桌椅” (desks-and-chairs) is derived jointly from each single morpheme comprising it. Following this observation, Hoosain [28] states that “the meaning of the constituents of polymorphemic Chinese words (multi-characters) are more manifest than often is the case with constituents of multimorphemic English words” (p.115). As such, it seems plausible that encoding and meaning retrieval of polymorphemic words in Chinese tends to be more rapid and prominent in Chinese literacy attainment [29]. To put it differently, lexical morphology seems to serve as a powerful device for meaning activation of polymorphemic vocabulary items, and thereby facilitating reading development through the construction of chunk meaning [30]. The semantic knowledge of constituent morphemes benefits the learners with the ease of computing the whole meaning of a Chinese compound. It is important to note that although collocation and compound words are composed of constituent characters, there is a fine line between compounds and collocations. Collocation refers to the phenomenon whereby a word usually retains company with other words [31]. It follows some selection restrictions of the syntactic and semantic relations running between the constituent characters, and the understanding of collocations is knowing which/what type of word co-occurs with the target word, and which/what type of word must be used with this one [32]. For instance, in Chinese, “两” and “双” means the same as “being more than one”, but the nouns they can match are restricted by semantic information. “两本书 (two books)”, “双月刊” (bimonthly journal)” are more acceptable than “双本书”, “两月刊”. “两” is used to describe things that are great in amount; while “双” is used to describe things made of equal or similar parts). It is subsumed under the facet of “use” in word knowledge [33]. Sensitivity to compound words is not constrained by the convention of the juxtaposition of words and it involves the ability to identify the center constituent of a compound word and to construct a new morphologically complex word from previously known morphemes [24].

### Morphological awareness in higher-order reading acquisition

#### Dimensionality of MA in reading acquisition

The significant associations between morphology and literacy outcomes are subject in part to the framing of the MA construct, which invites a deliberate scrutinization into the complexity and nature underlying morphological awareness (i.e. dimensionality). Recent studies explicitly probed into the latent factor structure underlying morphological awareness [6, 7], yielding mixed findings as to the complexity of morphological knowledge [34]. There is some evidence in support of multidimensionality of morphological knowledge in reading. Goodwin et al. [6] examined the dimensionality underlying morphological awareness among English-speaking seventh- and eighth- graders and the extent to which dimensions of morphological awareness contributed to vocabulary knowledge and reading comprehension skills. Confirmatory factor analysis underscored the multidimensionality of morphological knowledge that could be dissected into general morphological knowledge and task-related morphological dimensions. Intriguingly, the expected conceptual distinction between tacit/intuitive morphological processing and strategic morphological analysis were not confirmed in their study, which implied the intuitive ability to manipulate morphemes and explicit analysis of morphemic structure to infer the meaning of unfamiliar multimorphemic words fell into a unitary construct underlying morphology. More importantly, structural equation modeling confirmed the predictive validity of the bifactor model in both vocabulary knowledge and reading comprehension. Specifically, the general factor of morphological knowledge explained a significant proportion of variance in reading outcomes. Nonetheless, by examining seven different dimensions, the results yielded different predicting variances in reading. The specific measurement of morphological relatedness significantly predicted vocabulary and the only significant task supporting reading comprehension was morphological meaning processing, which suggested that general sensitivity to morphological structures and attention to morphological meanings establish crucial morphological regard for reading attainment. In addition to the multidimensionality of morphological awareness, the unidimensionality underlying morphological awareness was also supported by a number of recent empirical studies. For instance, Tibi and Kirby [7] confirmed the unidimensionality of morphological awareness using multiple morphological awareness measures varying in task requirements (oral vs. written, single word vs. sentence contexts, and standard vs. local dialect) among 3rd grade Arabic-speaking children. Their findings also confirmed an acceptable bi-factor model when task modalities were split into oral assessments and written assessments. Both one- and two-factor solutions explained a substantial amount of variance in word reading. Similarly, James et al. [35] explored morphological awareness and reading comprehension among three age groups of English-speaking children: aged 6 to 8 years, 9 to 11 years, and 12 to 13 years. Morphological awareness tasks included judgment and production tasks of different morphological structures (inflection, derivation, and compounding). The principal component analysis identified a single factor of morphological awareness across each age group. Additional regression analyses further verified their predictive validity in reading comprehension. Moreover, quantile regression analysis revealed a similar extent in reading comprehension explained by morphological awareness and vocabulary across the ability range. It indicated the stability of the validity of morphological awareness and vocabulary in reading development across different levels of readers.

The aforementioned literature seems to imply a universal construct of MA with regard to content and process; whereas the diversity of task modality may underlie the multidimensionality of MA. That is, explicit processing of morphemic information and strategic analysis of multimorphemic words are essentially single-dimensional and the underlying cognitive nature of MA is unitary. This account resonates with the definition of morphological awareness specifying the ability to recognize and manipulate morphemic structure [1]. Intriguingly, previous work also alluded to a close association between MA and vocabulary as well as the predictive validity of the two constructs in reading. It stands to reason since meaning can be retrieved during morphological analysis by decomposing unfamiliar words into constituent morphemes and subsequently vocabulary grows [36]. Also, morphological awareness is a critical indicator of vocabulary depth (e.g. [37]), and morphologically complex words contributed to 60% of the vocabulary for students above 4^th^ grade [38]. The mounting evidence on the close association between vocabulary and morphology motivated studies on the MA construct to investigate MA and vocabulary simultaneously. Some recent literature on children has confirmed a unidimensionality of MA and vocabulary, and considered MA to be an additional facet of readers’ vocabulary knowledge [8, 10]. Spencer et al. [8] examined the underlying dimensions of morphological awareness and vocabulary knowledge among elementary-aged English-speaking children using different morphological awareness tasks and incorporating breadth and depth of knowledge into vocabulary measurements. Their findings revealed that morphological awareness and vocabulary assessments were loaded onto one unitary factor.

In contrast, some studies on adults attest to the multidimensionality of MA separate from vocabulary. For instance, Tighe and Schatschneider [9] reported a three-factor CFA with separate dimensions of real-word morphological awareness, pseudoword morphological awareness and vocabulary knowledge and such a distinction provided the best fit for English reading comprehension. Building on this work, Tighe and Schatschneider [39] further analyzed the predictive power of different dimensions of morphological awareness and vocabulary knowledge to reading comprehension, and reported that the three-factor model of real word morphological awareness, pseudoword morphological awareness, and vocabulary knowledge was the best-fitting model to predict reading comprehension. Among the three factors, vocabulary knowledge was the only significant predictor of reading comprehension. The multidimensionality of morphological awareness separable from vocabulary supports that morphological awareness is distinct from vocabulary among adult students, which echoes the results of a longitudinal study [40] that latent factors of vocabulary knowledge and grammatical abilities (morphosyntactic knowledge) became increasingly more disassociated as grade level progressed (from kindergarten to grades 2, 4, and 8). The series of studies highlights the possible conceptual and methodological discrepancy between morphological awareness and vocabulary knowledge among mature readers.

In summary, there are distinct features underlying morphological awareness (e.g., oral versus written, real word versus pseudoword) and morphological awareness may be not distinct from vocabulary in young readers. Additionally, previous research has predominantly focused on the underlying construct and construct validity among alphabetic readers [8, 10] or partial phonemic/abjad readers [7]. Nonetheless, there is a paucity of studies testing the multidimensionality of morphological awareness and its relationship to vocabulary and higher-order reading comprehension among logographic readers. Since the different conceptualizations and operationalizations of morphological awareness may impact the understanding of the construct underlying morphological knowledge, the discrepancy between alphabetic and logographic scripts may underpin the variations in the complexity of the MA construct. Therefore, the dimensionality of morphological construct in logographic languages (i.e., Chinese) warrants examination.

#### Mediated and unmediated role of MA in reading comprehension

Evidence accumulated in the past decades converges on the strong association between morphological awareness and reading comprehension. Morphology conveys semantic, grammatical, and syntactic information [41]. It binds phonological, orthographic and semantic features of words [42]. The more encounters with morphemes and words in different contexts, the stronger lexical representations become in memory, subsequently the more proficient reading comprehension skills accumulate [43]. Morphological awareness has been implicated in the context of modeling skilled reading; however, there is no consensus as to the precise nature of the relation between MA and reading comprehension. A strand of research underpins the direct morphological pathways to reading comprehension across different languages [3, 17, 18]. These studies suggest that morphological awareness makes a unique and significant contribution to reading comprehension over and beyond skills such as vocabulary and word reading. For instance, drawing upon structural equation modeling, Kieffer and Lesaux [17] discovered that morphological awareness was directly correlated with reading comprehension after controlling for reading vocabulary and word reading fluency. In addition, their findings revealed an indirect contribution of morphological awareness to reading comprehension via the mediation of vocabulary, suggesting an indirect morphological pathway to reading. Following this line of inquiry, many researchers have investigated additional variables to disentangle this mediating mechanism by which morphological awareness contributes to reading comprehension, with several potential mediators being proposed: word reading [44], reading vocabulary [16, 17, 19], morphological analysis [45, 46], as well as lexical inferencing ability [16].

As argued in the Morphological Pathways Framework, morphological awareness can facilitate word reading [2]. Given that word reading is a vital component in the initial stage of learning to read [47], morphological awareness can contribute to reading comprehension through the mediation via accurate and efficient word reading. Moving beyond to higher-order reading processing, vocabulary knowledge is another proximal mediator. Supporting this view, a study of monolingual English-speaking children demonstrated a significantly indirect contribution of morphological awareness to reading comprehension via vocabulary knowledge [3]. In a study among Chinese-speaking English readers, Zhang and Koda [16] found no significant unique association between morphological awareness and reading comprehension, but a significantly indirect effect through the mediation of vocabulary knowledge. Moreover, they uncovered another indirect path route via the multiple mediations of lexical inferencing ability and vocabulary knowledge.

The indirect morphological pathway provided by the aforementioned study has underscored the importance of lexical inferencing ability. Lexical inferencing ability is conceptualized as “making informed guesses as to the meaning of a word, in light of all available linguistic cues in combination with the learner’s general knowledge of the world, her awareness of context and her relevant linguistic knowledge” [48]. More recent studies have demonstrated its intertwined relations with morphological analysis, in which inferring morphologically complex words is involved. To put it differently, morphological analysis enables learners to infer meanings of unknown words by decomposing morphologically complex words into meaningful constituent morphemes, which can distinguish between poor and skilled readers, as demonstrated in the study of Zhang and Shulley [45]. They found that typical readers outperformed poor comprehenders on the use of morphological analysis to infer the meaning of unknown words in reading. A multitude of empirical studies supported the mediated effects of morphological awareness upon reading comprehension via lexical inferencing ability or morphological analysis. Levesque et al. [49] conducted a study to probe into the indirect morphological pathways among English-speaking children. The results indicated that morphological awareness offered an indirect contribution to reading via morphological analysis. Building on this work, Levesque et al. [46] further evaluated the longitudinal predictive validity of morphological skills among English-speaking children from grade 3 to grade 4. Their findings revealed that morphological analysis, rather than morphological awareness, contributed to gains in reading comprehension from grade 3 to grade 4. Zhang, Lin, Liu, and Nagy [50] further investigated the mediation mechanism among students with varying English proficiency levels. The results showed that morphological analysis mediated the significant contribution of morphological awareness to reading comprehension. Moreover, students with higher-level English proficiency (native English speakers and proficient English learners) were more able to use morphological cues to infer the meanings of new words, which could in turn contribute to reading comprehension.

The robust relationship between lexical inferencing ability and reading comprehension has also been documented in Chinese-speaking children [19, 20]. Zhang [19] provided empirical evidence that morphological awareness contributed to reading comprehension via the mediation of lexical inference ability among Chinese-speaking second graders. Building on this work, Zhang [20] further examined the indirect morphological pathways among Chinese-speaking children at two time points with an interval of one semester. The results supported the association between morphological analysis and reading comprehension, indicating that lexical inferencing ability mediated the effect of morphological awareness upon reading comprehension concurrently and longitudinally.

The above literature clarifies both the direct and indirect morphological pathways to reading comprehension. It seems reasonable that the mediation of morphological analysis (contextual morpheme-based inference), but not morphological awareness, contributes more to reading comprehension [51]. However, the extent to which the above-mentioned mediators can facilitate reading across different languages is still underexplored. More evidence is thus required to ascertain the interplay between these mediators, and to specify their shared and unique contributions to reading comprehension.

## The current study

The first aim of the current study was to investigate whether morphological awareness was best represented as a single or multidimensional construct among Chinese early adolescent readers. Structural equation modeling was adopted to explore whether morphological awareness and vocabulary knowledge were separate constructs or the same underlying construct. Despite variations in morphological awareness measurements used to unravel the complexity of morphological construct, there has been no research accessing the distinctiveness of morphological awareness and vocabulary in Chinese early adolescent readers, and only a few relevant studies on the complexity of the two constructs have been conducted among English speakers of varying age groups from elementary-age children [8] to English-speaking adults [9, 39]. The uniqueness of lexical compounding in Chinese makes it compelling to study the construct of morphological awareness in logographic learners. Based on the previous study documenting morphological awareness as a unidimensional construct integral to vocabulary knowledge among children [8], we hypothesized that the factor structure of morphological awareness may also apply to early adolescent Chinese readers and that MA and vocabulary were better conceptualized as a unitary construct. Such assumption also stems from the theoretical notion that morphological awareness binds phonological, orthographic and semantic features of words [42], and morphological awareness was treated as an additional facet of vocabulary knowledge [8].

The second aim of the current study was to examine the relation between morphological awareness and higher-order reading comprehension. We hypothesized that the unitary dimension of vocabulary and morphological awareness contributed to reading comprehension via lexical inference based on the previous study highlighting the mediating role of lexical inference in the MA- reading comprehension association [16, 19, 20].

To summarize, there are two research questions addressed in the current study.

1. Is morphological awareness best represented as a single or multidimensional construct? Are morphological awareness and vocabulary knowledge separate constructs or the same underlying construct among Chinese early adolescent readers?
2. Does morphological awareness have direct or indirect effects on Chinese early adolescent reading comprehension through mediating factors?

## Methodology

The study was approved by the IRB office of East China Normal University (Protocol number: HR 182–2021) and was performed in accordance with the ethical standards as laid down in the 1964 Declaration of Helsinki and its later amendments or comparable ethical standards. Oral informed consent was obtained before the data collection.

### Participants

686 early adolescent students (339 Grade 5 students and 347 Grade 6 students, 389 boys and 297 girls, Mean age = 12.5 years) participated in the study. The participating students were from two public schools in Suzhou and Wenzhou, China and they were mostly from working-class families. They started to have intensive literacy instruction from the first grade. The participating schools were from two coastal provinces, Jiangsu and Zhejiang. Their curricula were stipulated by the Bureaus of Education in respective provinces, which also followed the guidelines of the criteria proposed by the Ministry of Education of the People’s Republic of China [52]. By the late elementary age, students should be able to recognize familiar Chinese characters rapidly; read passages fluently; comprehend passages while reading aloud; understand sentences with complex syntactic structures; extract core textual information; understand structural and logical connections between paragraphs; and understand main ideas.

### Test Battery

#### Morphological awareness

Students’ morphological awareness was measured by compound structure awareness, morpheme discrimination and morpheme recognition tasks. All prompt words in the recognition and discrimination tasks were high-frequency words and/or selected from the Chinese curriculum to ensure the items did not extend beyond the students’ vocabulary knowledge.

*Compound structure awareness*. Compound structure awareness assessed the children’s ability to understand compounding structures in Chinese word formation. The measurement was designed based on Chen et al. [24] and Liu and McBride-Chang [15]. Children were required to select the plausible compound word according to each prompt. The target compounds were low-frequent or novel words in real life to control for the possible familiarity effects of collocations, i.e. 云花(cloud flower), 冻金(frozen gold). In accordance with the four categories tested in Liu and McBride-Chang’s [15] study, four Chinese compounding structures were measured in the study: coordinate, subordinate, subject-predicate, and verb-object. For example, a prompt 星星在闪烁叫做什么? (How would you say “a star is twinkling”) was shown to the participants. They were asked to judge whether the target compound was 闪星(twinkling star) or 星闪(star twinkling). There was a total of 20 items in this measurement. The reliability of this measure was acceptable (Cronbach’s α = .749, McDonald’s ω = .795).

*Morpheme discrimination*. The morpheme discrimination task asked the participants to judge the morphemic outlier among three presented words. For example, 读者(reader), 作者(writer/author) and 或者(or) were shown to the students, one of which does not convey the morphemic meaning 者(-er/-or). The participating students were supposed to circle the odd man out. There were 20 items in this measure. The reliability of this measure was acceptable (Cronbach’s α = .734, McDonald’s ω = .779).

*Morpheme recognition*. The morpheme recognition measurement tapped into the students’ ability to understand the morphemic relationship between two words. The students were asked to judge the semantic relationship between a disyllabic word and its subcomponent. For instance, 海报(sea+paper→flyer) and its component word海(sea) were presented to the participants and they needed to judge whether these words are semantically related. There were 20 items in this measure. The reliability of this measure was acceptable (Cronbach’s α = .725, McDonald’s ω = .773).

#### Vocabulary knowledge

A vocabulary checklist task [53, 54] assessed children’s reading vocabulary knowledge. The participating students were asked to circle the words they knew. A total of 80 two-character words was presented to the participants. Among them, there were 64 real words and 16 non-words. Non-words consisted of real characters but the combination of the two characters were not legitimate in Chinese. For example, 可国 was one of the non-words, both component characters, 可(able) and 国(country) are real characters, however, the combination of the two does not exist Chinese. In data analysis, real words selected as known were coded as “real hits” and nonwords selected as known were coded as “false alarm”. According to the Signal Detection Theory, their scores were computed by *true h* = $\frac{h-f}{1-f}$ (h: real hits; f: false alarm).

#### Lexical inference ability

The lexical inference task was adopted from Zhang [19] and measured the children’s ability to form lexical meanings based on the morphemic cues (suffixes) within bimorphemic words. Suffixes provided clues to the word class and meaning of the entire compound word. For instance, a bimorphemic word笔直(perfectly straight/upright) was shown to the participants and they needed to choose the most appropriate meaning explanation from the following options: 把笔借给同学(lend pen to classmates), 一只直的笔(a straight pen), 像笔杆一样直(straight like a pen), and 不可以转弯 (cannot make a turn). The participants were supposed to choose the third option if they knew the morphemic meaning of “直(straight)” and the compound structure of a modifier. There are 20 questions in this measure and the reliability was acceptable (Cronbach’s α = .820, McDonald’s ω = .868).

#### Reading comprehension

The reading comprehension task measured participants’ abilities to identify specific textual information, make text-based inferences, and interpret the gist of each passage. There was a total of five passages including narrative and expository texts. Three to five comprehension multiple-choice questions were presented after each text. The questions consisted of gist detection, specific information identification, and text-based inference. For example, the participants were asked to infer text information, “what does this information indicate?” and to understand the main idea of the text, “what is the main idea of the passage?”. There were 18 questions in total and each question was worth 1 point. The reliability of this measure was acceptable (Cronbach’s α = .725, McDonald’s ω = .776)

#### Procedure

All measurements were paper-and-pencil tests and completed during school hours in quiet classrooms. Each task was administered to the participants in a separate week, and the order of the tasks was counterbalanced to rule out priming or carry-over effects from previous tasks. There were two sessions of testing, first being implemented in Suzhou and then in Zhejiang. The total time allotment for all measurements in each session was about 70 to 80 minutes. And the collection procedure was completed within 5 weeks in each school.

### Data analysis

We performed a pattern of path models using Amos 23.0 to explore the dimensionality of MA and vocabulary as well as the plausible pathways from MA to higher-order reading skills. To test our hypothesis that morphological awareness and vocabulary may be a unitary construct, we formulated three sets of path models to evaluate the complexity of morphological awareness and subsequently the possible mediating effects. The first path model was established in which morphological awareness measurements were treated as exogenous variables, and they contributed to reading comprehension through the mediation of both vocabulary and lexical inference. The second path model was conducted in which morphological awareness contributed to vocabulary, which then contributed to lexical inference, and subsequently, contributed to reading comprehension. The third path model was performed in which morphological awareness and vocabulary were treated as parallel covariates, and together contributed to reading comprehension through lexical inference. The goodness of the fit for the proposed models was evaluated using chi-square values and a set of fit indexes as follows: (a) the Comparative Fit Index (CFI) and Normed-fit Index (NFI), Non-Normed Fit Index (NNFI) scaled over .95 signifying a good fit [55]; (b) the Root Mean Square Error of approximation (RMSEA) values less than 0.08 indicating good-fitting models [56]. Note that the sample size in the current study amounted to 686, far above the previously published minimum of *N* = 200 for analytic variable modeling [57]. Hence, the current sample size is sufficiently large to offer a precise estimate of goodness of fit. Significance was determined using a cut-off point of *p* < .05.

## Results

### Descriptive statistics and correlations

The descriptive analysis in Table 1 presents the mean, range and standard deviation of each measurement. In general, there was adequate variability across the measured abilities. A relatively wide dispersion was found in the vocabulary measurement given that raw scores were converted to percentage rates. Among the measurements, the morpheme discrimination task had the highest accuracy rate (91.2%) while the reading comprehension measurement had the lowest accuracy rate (60.6%). Table 2 presents the Pearson correlation coefficients of the variables. Age had no or weak correlations with all the tested variables. Morphological awareness measurements had significant correlations with vocabulary knowledge, lexical inference and reading comprehension (*r* = .173, *p* < .001 to *r* = .330, *p* < .001). Vocabulary knowledge also had significant correlations with lexical inference and reading comprehension (*r* = .284, *p* < .001; *r* = .311, *p* < .001; *r* = .316, *p* < .001).

**Table 1:** Descriptive statistics of morphological awareness, vocabulary knowledge, lexical inference and reading comprehension.

Variables	Min	Max	M (Percentage)	SD
Compound awareness (20)	8	20	15.40 (77.0%)	0.60
Morpheme recognition (20)	6	20	15.81 (79.1%)	2.65
Morpheme discrimination (20)	4	20	18.23 (91.2%)	2.10
Vocabulary knowledge (100)	20	100	82.62 (82.6%)	19.40
Lexical inference (20)	1	19	13.30 (66.5%)	2.32
Reading comprehension (18)	1	18	10.90 (60.6%)	2.67

*N* = 686. Numbers in the parentheses represent the maximum score of each measurement.

**Table 2:** Bivariate correlations among morphological awareness, vocabulary, lexical inference and reading comprehension.

Variables	1	2	3	4	5	6	7
1. Age	-						
2. Compound awareness	.074	-					
3. Morpheme recognition	.133***	.242***	-				
4. Morpheme discrimination	.053	.173***	.281***	-			
5. Vocabulary knowledge	-.083*	.223**	.330***	.304***	-		
6. Lexical inference	.109**	.200***	.326***	.304***	.311***	-	
7. Reading comprehension	.116**	.182***	.271***	.216***	.284***	.316***	-

*p < .05

**p < .01

***p < .001

### Estimation of structural models

**Fig 1:**
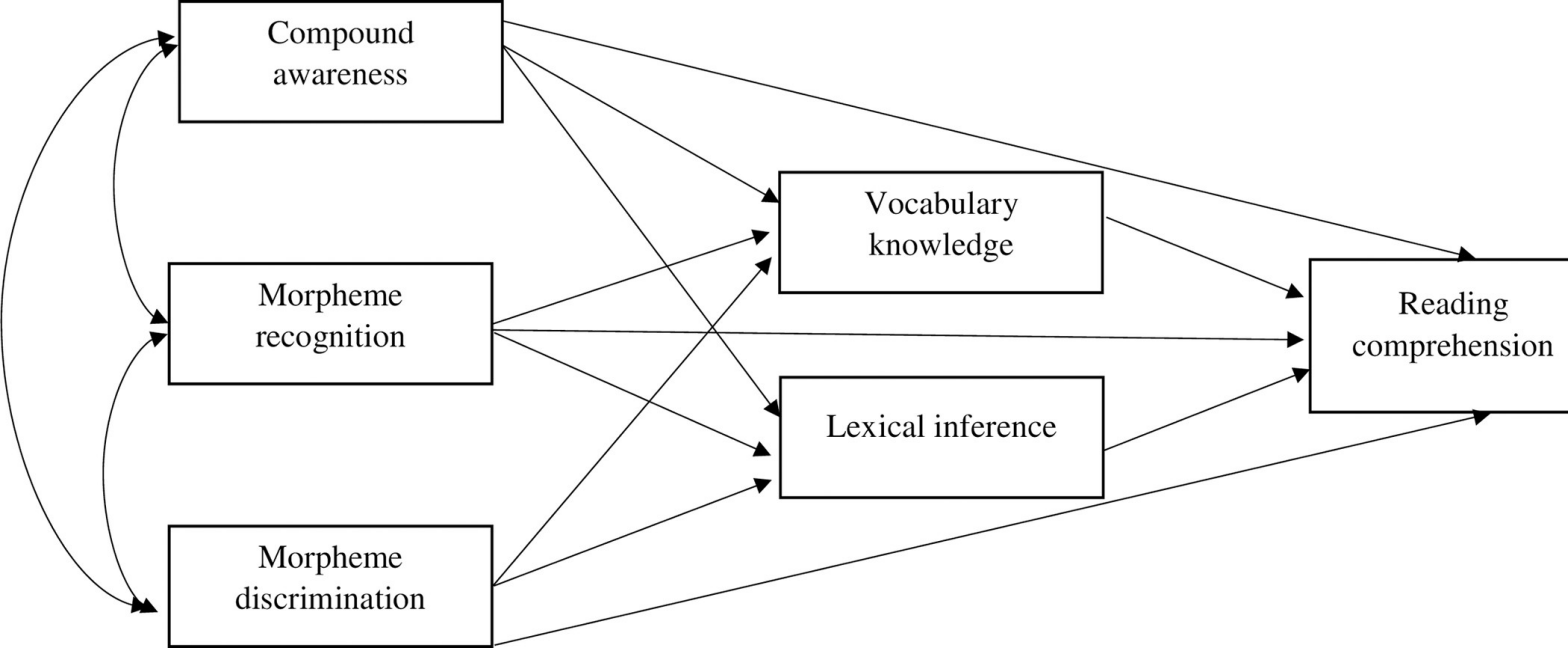
Model 1 showing multidimensionality of morphological awareness separate from vocabulary knowledge in predicting reading comprehension via the mediation of both vocabulary knowledge and lexical inference.

**Fig 2:**
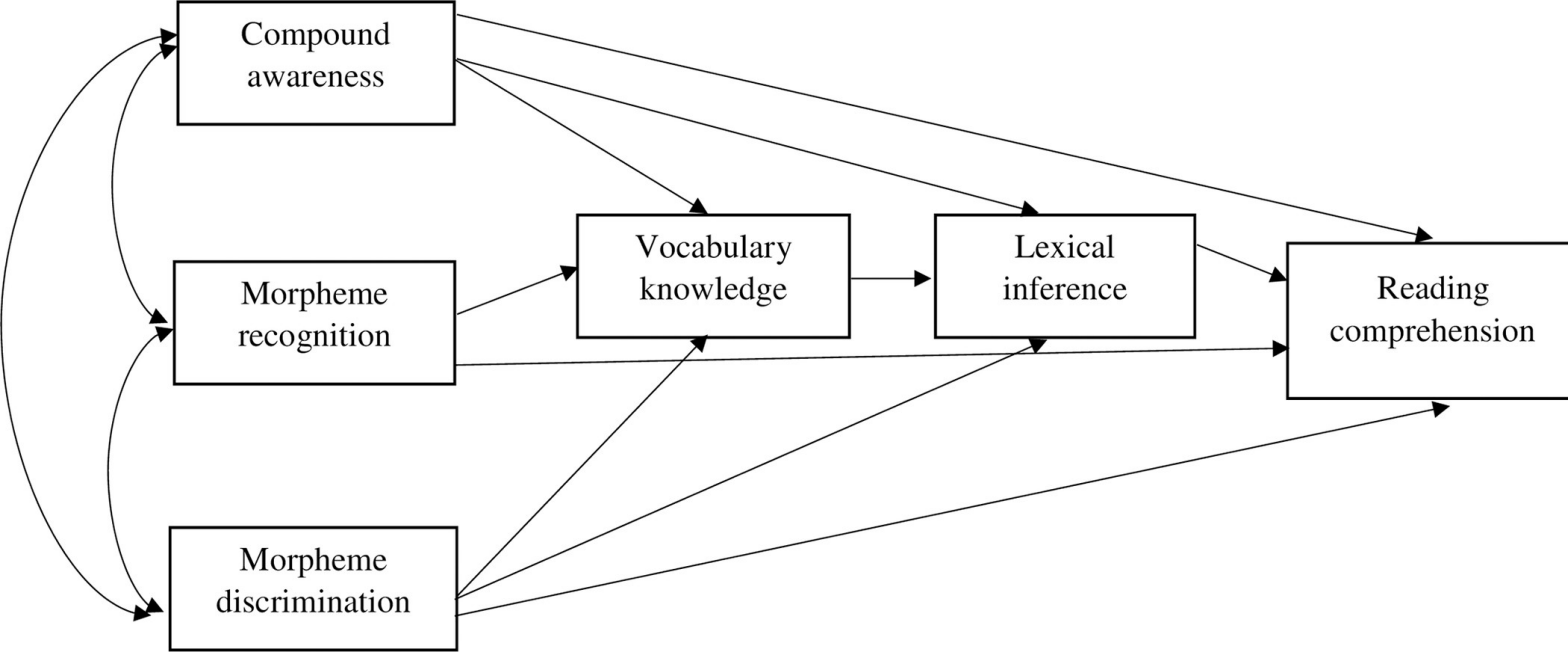
Model 2 showing multidimensionality of morphological awareness separate from vocabulary knowledge in predicting reading comprehension via the path from vocabulary knowledge to lexical inference.

**Fig 3:**
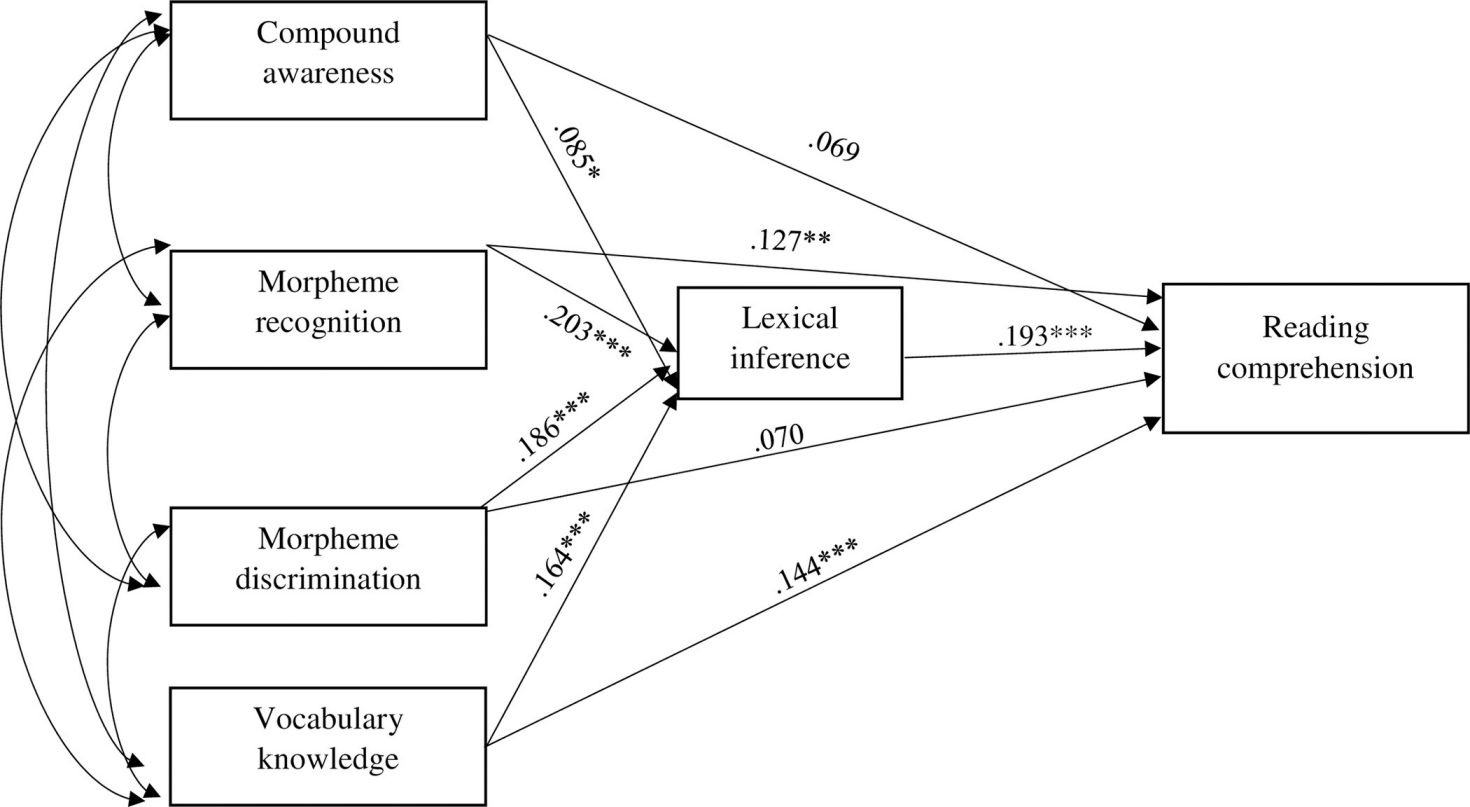
Model 3 showing the unidimensionality of morphological awareness and vocabulary knowledge in predicting reading comprehension via lexical inference. Note * *p* < .05, ** *p* < .01, *** *p* < .001.

A number of path models were formulated to test the dimensionality of morphological awareness and vocabulary, and subsequently analyzed the mediating effect. Fig 1 presents the first model that assumes morphological awareness and vocabulary knowledge are two separate constructs, in which vocabulary knowledge and lexical inference both mediates the relationship between morphological awareness facets and reading comprehension. Similarly, Fig 2 presents the second model that hypothesizes the separate constructs of morphological awareness and lexical inference. However, lexical inference is hypothesized to have multiple mediating routes: mediation between morphological awareness and vocabulary knowledge, mediation between vocabulary knowledge and reading comprehension, and mediation between morphological awareness and reading comprehension. Finally, Fig 3 shows the path model assuming that vocabulary knowledge and morphological awareness underlie the same construct which contributes to reading comprehension through lexical inference.

Table 3 shows the results of model fit indices thus identifying the best-fitting model to the research questions. The extant psychometric literature suggests that Comparative Fit Index (CFI), Normed-fit Index (NFI), Non-Normed Fit Index (NNFI) greater than .95 [55] and a Root Mean Square Error of Approximation (RMSEA) less than 0.08 [56] show a good model fit. Based on the model fit indices, the third model hypothesizing the same construct of morphological awareness and vocabulary knowledge had the best model fit.

**Table 3:** Model fit indices of alternative path models.

Model	*χ* ^2^	*df*	*p*	*χ*^2^/*df*	RMSEA	CFI	NFI	NNFI
1	17.80	1	.000	17.80	.158	.964	.964	.966
2	7.04	1	.008	7.04	.094	.987	.986	.988
3	3.62	1	.057	3.62	.062	.994	.993	.995

### Testing mediated and unmediated effects

Table 4 presents the results of regression weights of the best-fitting path model. All morphological awareness facets (morpheme recognition, morpheme discrimination and compound awareness) made significant contributions to lexical inference ($\hat{\beta }$ = .203, *p* < .001, $\hat{\beta }$ = .186, *p* < .001, $\hat{\beta }$ = .085, *p* < .05). Of all the morphological awareness measurements, morpheme recognition had a significant effect on reading comprehension ($\hat{\beta }$ = .127, *p* < .005) while morpheme discrimination and compound awareness had marginal or nonsignificant effects on reading comprehension ($\hat{\beta }$ = .070, *p* = .070, $\hat{\beta }$ = .069, *p* = .064). Vocabulary knowledge and lexical inference had significant impacts on reading comprehension ($\hat{\beta }$ = .193, *p* < .001, $\hat{\beta }$ = .144, *p* < .001).

**Table 4:** Standardized regression weights for the preferred path model.

Paths			$\hat{\beta }$	*S*.*E*.	*C*.*R*.*(z)*	*p*	*95% CI*
LEXI	<---	MORR	.203	.033	5.380	.000	[0.138, 0.268]
LEXI	<---	MORD	.186	.043	4.992	.000	[0.101, 0.270]
LEXI	<---	VOCK	.164	.456	4.284	.000	[-0.731, 1.059]
LEXI	<---	COMS	.085	.040	2.330	.020	[0.006, 0.164]
READ	<---	LEXI	.193	.045	4.957	.000	[0.105, 0.281]
READ	<---	MORR	.127	.040	3.234	.001	[0.048, 0.206]
READ	<---	MORD	.070	.052	1.813	.070	[-0.032, 0.172]
READ	<---	VOCK	.144	.542	3.651	.000	[-0.920, 1.208]
READ	<---	COMS	.069	.047	1.851	.064	[-0.023, 0.161]

*MORR* Morpheme recognition; *MORD* Morpheme discrimination; COMS Compound awareness; *VOCK* Vocabulary knowledge; *LEXI* Lexical inference; *READ* Reading comprehension

To further test the mediated and unmediated effects of morphological awareness on reading comprehension, direct and indirect effects as well as total effects were analyzed (Table 5). The results confirmed the significant collective effect of all variables on reading comprehension given the significant total effects. However, no direct effect was found between the variables and reading comprehension. Given the significant direct effect of lexical inference ($\hat{\beta }$ = .193, *p* < .001), all variables were mediated by lexical inference. In conjunction with the results presented in Table 5, the findings showed that vocabulary knowledge, morpheme recognition and compound structure awareness contributed to reading comprehension both directly and indirectly via lexical inference. Morpheme discrimination contributed to reading comprehension indirectly through lexical inference.

**Table 5:** Standardized total effects, direct effect, and indirect effect on reading comprehension.

Reading Comprehension	Direct Effect	Indirect Effect	Total Effects
Vocabulary knowledge	.144***	.032	.176***
Morpheme discrimination	.070	.036	.106**
Morpheme recognition	.127**	.039	.166**
Compound awareness	.069	.016	.085*
Lexical inference	.193***	-	.193***

*p < .05

**p < .01

***p < .001

## Discussion

### Dimensionality of morphological awareness and vocabulary knowledge

The first research question addressed whether morphological awareness and vocabulary knowledge represented a multidimensional construct in Chinese reading. After testing multiple alternative path models, the study showed that morphological awareness and vocabulary knowledge were best represented as parallel covariates in the model of Chinese reading comprehension. In other words, morphological awareness and vocabulary knowledge appeared to be under the same construct contributing to Chinese reading comprehension. As discussed above, there have been mixed findings regarding the dimensionality of morphological awareness and vocabulary knowledge. One strand of research stressed the multidimensionality of morphological awareness and the predicting power of morphological awareness in vocabulary knowledge [6, 9] while the other strand of research highlighted the unitary construct of morphological awareness and vocabulary knowledge [8, 37]. The current study found that morphological awareness and vocabulary knowledge appeared to be parallel components under the same construct, which collectively contributed to reading comprehension.

The commonality underlying morphological awareness and vocabulary knowledge is an important question to be further explicated. Our interpretation is in line with the categorization of word-specific knowledge and word-general knowledge [58]. Word-general knowledge includes morphological awareness that monitors partial-word structures and meanings. The fundamental utilities of morphological awareness are to decompose complex structures and to build semantic links among lexical bundles. Similarly, the essential role of vocabulary is to build semantic connections in larger chunks (sentence or discourse). Given the commonality between morphological awareness and vocabulary knowledge, both components are likely to fall on the unitary construct of word knowledge. Li and Kirby [37] also verified that morphological awareness was an indicator of vocabulary knowledge. Another interpretation of the results lies in the language-specificity of measured morphological awareness. English orthography is a morphophonemic writing system with alphabetic letters as the basic unit of writing which entails phonemic and morphological properties [59]; while Chinese orthography is a logographic and morphosyllabic writing system in which one individual character encodes one morpheme (syllable). The fundamental building blocks of Chinese words are morpheme-based characters. Early elementary-age children’s word learning starts from the initial decoding and decomposition of morpheme (character) meanings. Gradually, Chinese children need to learn and memorize multiple-character morphologically complex words. Therefore, morphological decomposition is integral to adolescent readers’ vocabulary. The current study highlighted morphological meaning activation and compounding structure awareness, which are requisites of vocabulary knowledge.

### Morphological awareness in reading comprehension: Mediation through lexical inference

The second research question addressed the direct and indirect effects of morphological knowledge on Chinese reading comprehension. Prior studies have shown a unique contribution of morphological awareness to reading comprehension among monolingual English-speaking children [3] and young English language learners [58]. Similarly, the current study yielded that morphological awareness predicted reading comprehension among Chinese adolescent readers. Morphological awareness can facilitate the integration of word representations by strengthening the relations between phonology, orthography, and semantics and subsequently can enhance representational qualities. Skilled reading comprehension can arise from high-quality word representations [42, 43]. However, it should be noted that the direct effect of morphological awareness on reading comprehension was relatively marginal compared with the mediated effect through lexical inference. To be specific, among all morphological awareness facets, only morpheme recognition had a significant direct effect on reading comprehension while the direct effect of morpheme discrimination and compound awareness was non-significant. Furthermore, a close inspection of the results revealed a significant direct effect of lexical inference on reading comprehension. We, therefore, would argue that the shared variance between morphological awareness and reading comprehension was largely attributed to the mediating role of lexical inference. To put it simply, our finding highlighted an indirect lexical inference pathway associating morphological awareness and reading comprehension.

The mediating role of lexical inference is in accordance with previous L1 and L2 research that examined the mediating variables associating morphological awareness and reading comprehension [16, 20]. Zhang and Koda [16] found that lexical inferencing skill was a critical mediator between morphological awareness and reading comprehension among adult EFL learners. Zhang [20] similarly highlighted the mediating role of lexical inference ability in reading comprehension among Chinese early elementary students. In line with this argument, we observed the path route from morphological awareness to lexical inference, and ultimately to reading comprehension. The path indicated that morphological awareness enhanced the children’s ability to form lexical meanings and infer meanings of unknown words, which in turn facilitated their reading comprehension. However, it is important to consider why lexical inferencing mediated the relationship between morphological awareness and reading comprehension. To begin with, it is worth mentioning that the notion of lexical inference ability in the current study builds on the ability to successfully infer meanings of unfamiliar words by using intra-word information and contextual information. The facilitative impact of lexical inference ability underlined its twofold functions in meaning retrieval: the integration of word-internal (morphology) and word-external (context) facets. Lexical inference ability centers on contextual clues constructed in context when learners read and make informed guesses based on given texts. Therefore, the indirect pathway associated word knowledge (morphological awareness and vocabulary knowledge) and reading comprehension. The current study focused on reading comprehension tasks that tapped into participants’ abilities to locate textual information and interpretation of main ideas. In this regard, students’ reading comprehension skill was largely contingent upon the use of context, which may account for the minimal contribution of morphological awareness to reading comprehension. More important, another possible explanation is to consider the internal feature of lexical inferencing ability, which is morphology-based. As a result, students, who possess better morphological awareness, tend to infer unfamiliar morphologically complex words better by using intra-word morphological cues, and subsequently achieve better comprehension. Our conceptualization of lexical inference resonates with the utilities of morphological analysis in reading comprehension [45, 46, 49, 51]. Morphological analysis pertains to the ability to analyze the meaning of a morphologically complex but unfamiliar word based on its morpheme constituents. Moreover, in an array of articles investigating morphological pathways in reading, Levesque et al. [49, 51] found that morphological analysis, but not morphological awareness, was a predictor of reading comprehension. In this respect, our finding reinforced that morphological analysis, or morpheme-based lexical inference ability, significantly mediated the contribution of morphological awareness to reading comprehension. Our study suggests that lexical inference (contextual morpheme-based inference) is an important predictor associating morphological awareness and reading comprehension among Chinese adolescent students.

Collectively, the current study found that morphological awareness and vocabulary knowledge were best represented as parallel covariates underlying the same construct. Furthermore, the study emphasized the mediating role of lexical inference in word knowledge and reading comprehension among Chinese adolescent readers.

## Conclusion

Although future research is needed on this front, e.g. using a longitudinal design, the current study provides a step towards the complexity of Chinese morphological awareness by unravelling the unidimensional construct of MA and vocabulary contributing to higher-order reading comprehension via lexical inference in an understudied population of early adolescent Chinese learners. MA and vocabulary may represent a shared construct and are possibly subsumed under a unitary dimension of word-level knowledge. The contribution of such word knowledge to higher-order reading ability was manifested through their influence on lexical inference. These findings have important pedagogical implications for incorporating vocabulary knowledge and dimensions of morphological awareness into instruction practices in learning to read among early adolescent Chinese learners. Moreover, inferencing ability should be made an important feature in instruction given its role in bridging the connection between MA and reading comprehension.

## Supporting information

S1 Dataset (SAV)
